# CARS spectroscopy of *Aspergillus nidulans* spores

**DOI:** 10.1038/s41598-018-37978-6

**Published:** 2019-02-11

**Authors:** Benjamin D. Strycker, Zehua Han, Blake Commer, Brian D. Shaw, Alexei V. Sokolov, Marlan O. Scully

**Affiliations:** 1 Institute for Quantum Science and Engineering, Texas A&M University, College Station, Texas USA; 2 Baylor University, Waco, Texas USA; 3 Department of Plant Pathology and Microbiology, Texas A&M University, College Station, Texas USA

## Abstract

Coherent Anti-Stokes Raman Spectroscopy (CARS) is performed on single spores (conidia) of the fungus *Aspergillus nidulans* in order to establish a baseline measurement for fungal spores. Chemical maps of single spores are generated and spectral differentiation between the cell wall and the cytoplasm is achieved. Principal Component Analysis of the measured spectra is then completed as a means to quantify spore heterogeneity. Applications range from the quick and accurate diagnosis of public health concerns to real-time agricultural and environmental sensing of fungal symbionts and pathogens.

## Introduction

Fungi (such as molds) are ubiquitous both in indoor and outdoor environments, and their actions upon human health and activity can be costly. Harmful molds that grow within homes and buildings can produce mycotoxins that poison the human body and exacerbate preexisting conditions^1,2^. Within the United States in recent years, mold-related asthma alone has affected approximately 4.6 million people for a total annual cost of $3.5 billion^3^. The agricultural losses from mold damage are also substantial and are estimated to be in the millions of dollars annually for US agri-producers^4^. Consequently, deleterious molds and mycotoxins pose a significant threat to public and economic health and have a large human toll^5^.

Minimizing the consequences of mold exposure and damage requires timely and accurate identification of mold species. Fungi are most commonly identified either by characterizing morphological features using light microscopy or by sequencing of housekeeping genes and comparing to genetic repositories^6,7^. Both of these techniques are time consuming and require considerable training for accurate identification. In contrast, Raman spectroscopy of collected mold spores has been proposed as a method to identify fungal species within several minutes. Ghosal *et al*. have used spontaneous Raman microspectroscopy to measure the spectra of seven mold species and differentiate them from one another^8^. Similarly, Farazkhorasani measured and differentiated between the spontaneous Raman spectra of several strains of *Aspergillus nidulans*^9^. Surface-Enhanced Raman Spectroscopy (SERS) of the mold species *A*. *nidulans* has also been reported^9,10^, although the highly-local nature of the field enhancement in this technique is more suited to detecting individual molecules than the complex mixture of chemicals that uniquely characterizes the spore of a fungal species and allows it to be identified.

A technically powerful counterpart to spontaneous Raman spectroscopy is Coherent Anti-Stokes Raman Spectroscopy (CARS), which possesses several advantages over spontaneous Raman. The spontaneous Raman signal intensity ${I}_{S}$ is proportional to the number of target molecules *N* such that ${I}_{S}\,\propto \,N$. The CARS process, however, induces a molecular coherence, resulting in a signal intensity ${I}_{{CARS}}$ that is proportional to the square of the number of target molecules, such that ${I}_{{CARS}}\propto {N}^{2}$. Consequently, the molecular coherence of the CARS process is able to provide a comparable signal-to-noise ratio to a spontaneous Raman process using integration times that are faster by at least two orders of magnitude^11^.

There is a large body of literature documenting the use of CARS in characterizing biological tissues and cells. Sophisticated, optimized schemes such as FAST-CARS have received significant attention in the application of quick and accurate identification of bacterial endospores to combat the threat of biological terrorism, even achieving single-pulse spectral characterization^12–14^. To date, however, no coherent Raman technique has been applied to the study of mold spores. In this paper we report the first such demonstration of CARS, a two-pulse broadband scheme applied in particular to the rapid spectral analysis and characterization of spores (conidia) of the fungus *A*. *nidulans*. We envision that, in the future, the technique will be applied to real-time identification of fungal spores and mycotoxins in domestic, commercial, and agricultural settings, thus addressing the pressing public health and economic needs that continue to be so costly.

## Materials and Methods

The *A*. *nidulans* wild-type strain FGSC A4^15^ was grown on minimal medium, prepared as previously described^16^, and incubated at 30 °C under continuous light. After 7 days, cultures were flooded with 2 mL sterile water and scraped with a sterile glass rod to release conidia (hereafter called spores). The spore suspension was washed at least three times by centrifugation (1 min. at 13,000 rpm) and stored in 1 mL sterile water at 4 °C. For experiments designed to examine cell walls of spores, 200 mg autoclaved glass beads (0.5 mm, Bio-Spec, Bartlesville, OK) were added to a subset of the sample and cells were disrupted for 15–20 s using a tissue homogenizer (Mini-Beadbeater, BioSpec, Bartlesville, OK).

To deposit the spores upon a microscope glass slide for subsequent CARS analysis, 8.7 μL of the spore suspension was pipetted onto the glass slide and evaporated at room temperature for several hours until only the spores remained. No spores that remained upon the glass slide for more than 24 hours were analyzed; new spore samples were pipetted from the suspension for each day’s experiments, so that the moisture content of the spores used was held constant.

**Figure 1:**
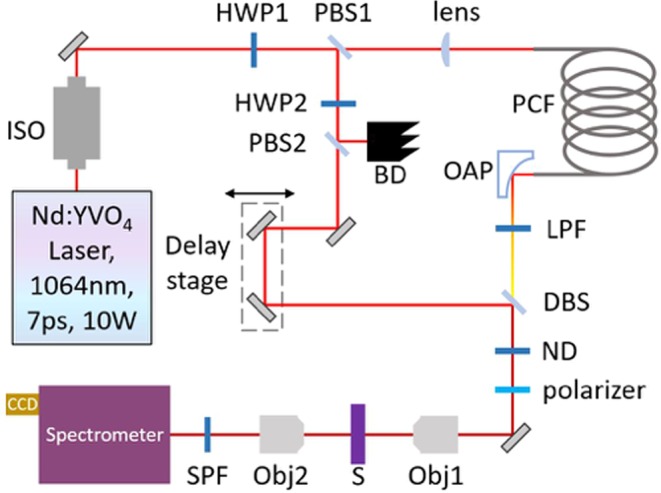
Schematic diagram of the experimental setup. ISO: isolator; HWP: half-wave plate; PBS: polarizing beam-splitter; PCF: photonic crystal fiber; OAP: off-axis parabolic mirror; LPF: long-pass filter; BD: beam dump; DBS: dichroic beam-splitter; ND: neutral density filter; Obj: microscope objective; S: sample; SPF: short-pass filter.

The experimental layout shown in Fig. 1 is based on that of Yujie Shen and coworkers^17^. A Nd:YVO_4_ laser (Attodyne Inc, APLX-10) produced pulses 7 ps in duration at 1064 nm with a repetition rate of 1 MHz. After passing through an isolator (Thorlabs, IO-5-1064-VHP), the output pulses were divided into two arms. Two pairs of half-wave plates and polarizing beam-splitters were used to control the power in the two arms, respectively. One arm, which contained a delay stage, served as the pump/probe beam. The other beam was converged by a lens (Thorlabs, LA1509-C) into a 2-m-long photonic crystal fiber (NKT Photonics, LMA-20) to generate broadband Stokes pulses, and then subsequently collimated by an off-axis parabolic mirror (Thorlabs, MPD129-P01). The Stokes beam was then propagated through a long-pass filter (Thorlabs, FELH1150) to remove shorter wavelength components before re-combination with the pump/probe beam through a dichroic beam splitter (Semrock Inc, LPD02-1064U-25). The delay stage was varied to control the temporal overlap between the two beams, and a linear polarizer (Thorlabs, LPNIR100-MP) was employed to maintain identical polarization. Subsequently, the two combined beams were focused by a 40X reflective objective (Thorlabs, LMM-40X-P01, 0.5 N.A.) and spatially overlapped on the sample. Both pump/probe and Stokes beams had a laser spot size approximately 2 μm in diameter, yielding a lateral spatial resolution of about 1 μm (see Appendix 1 in Supplementary Information), which was also the minimum step size of the motorized stage (Prior Sci, ES111) upon which the sample was mounted.

In addition to providing a much stronger signal compared to the spontaneous Raman setups of both Ghosal *et al*^8^. and Farazkhorasani^9^, our CARS microspectroscopy apparatus was transmissive. The CARS signal was collected by a 20X microscope objective (YSC Technologies, Plan Fluor, 0.45 N.A.) in the forward direction, followed by a short-pass filter (Thorlabs, FESH1000) and then sent into a spectrometer (Andor Inc, Holospec) to be detected by a CCD (Andor Inc, iDus416). All CARS spectra were taken using a beam power of 1.65 mW for the pump/probe beam and 0.95 mW for the Stokes beam, as measured with a powermeter (Thorlabs, S175C). See Appendix 2 in Supplementary Information for a characterization of the measured CARS spectra using different beam powers as a function of time.

Multiple CARS spectra were taken from each intact spore (*n* = 99) and from lysed spores (*n* = 17). Because of the biological variability of the spores, the non-resonant background of each measured CARS spectrum varied considerably. To correct for this large variation and to provide a standard of comparison for the measured spectra, the non-resonant background of each spectrum was subtracted with a 9^th^ order polynomial algorithm (*subback* function, based upon^18^, of the Biodata toolbox for MATLAB^19^) and then normalized by the subsequent CARS spectrum of the glass microscope slide upon which the fungal spores were deposited. Spectral normalization by the CARS spectrum of the glass substrate corrects for the varying spectral intensity of the supercontinuum probe pulse, under the condition that the glass substrate has no Raman resonances over the wavenumber range of the measurement (which is indeed the case here)^20–22^. The corrected CARS spectra therefore contain minimal contribution from the glass microscope slides and are primarily representative of the spores only. Finally, each resulting CARS spectrum was normalized with respect to the area under the curve.

For a subset (*n* = 48) of the intact spores mentioned above, the minimum diameter was able to be measured using image analysis software (ImageJ), calibrated with a resolution test target (R1DS1N, Thorlabs). In order to determine the relationship of spore diameter to measured CARS spectral variation, Principal Component Analysis (PCA) of the corrected and averaged CARS spectra of the edge and center of the spores was conducted. The average spectrum of the edge and center of each spore was calculated using the spectra corresponding to a radius of 1.2 and 0 μm, respectively. From the average spectrum (edge or center, respectively) of each of the *n* = 48 spores, a spectral correlation matrix was constructed using the Pearson correlation coefficient. The eigenvector corresponding to the largest eigenvalue of the matrix was then used to calculate the Principal Component (PC) score of each corrected spectrum.

Additionally, several CARS spectra of intact spores were taken using a sapphire substrate and, besides normalizing by the peak amplitude, were not processed in any way.

## Results and Discussion

**Figure 2:**
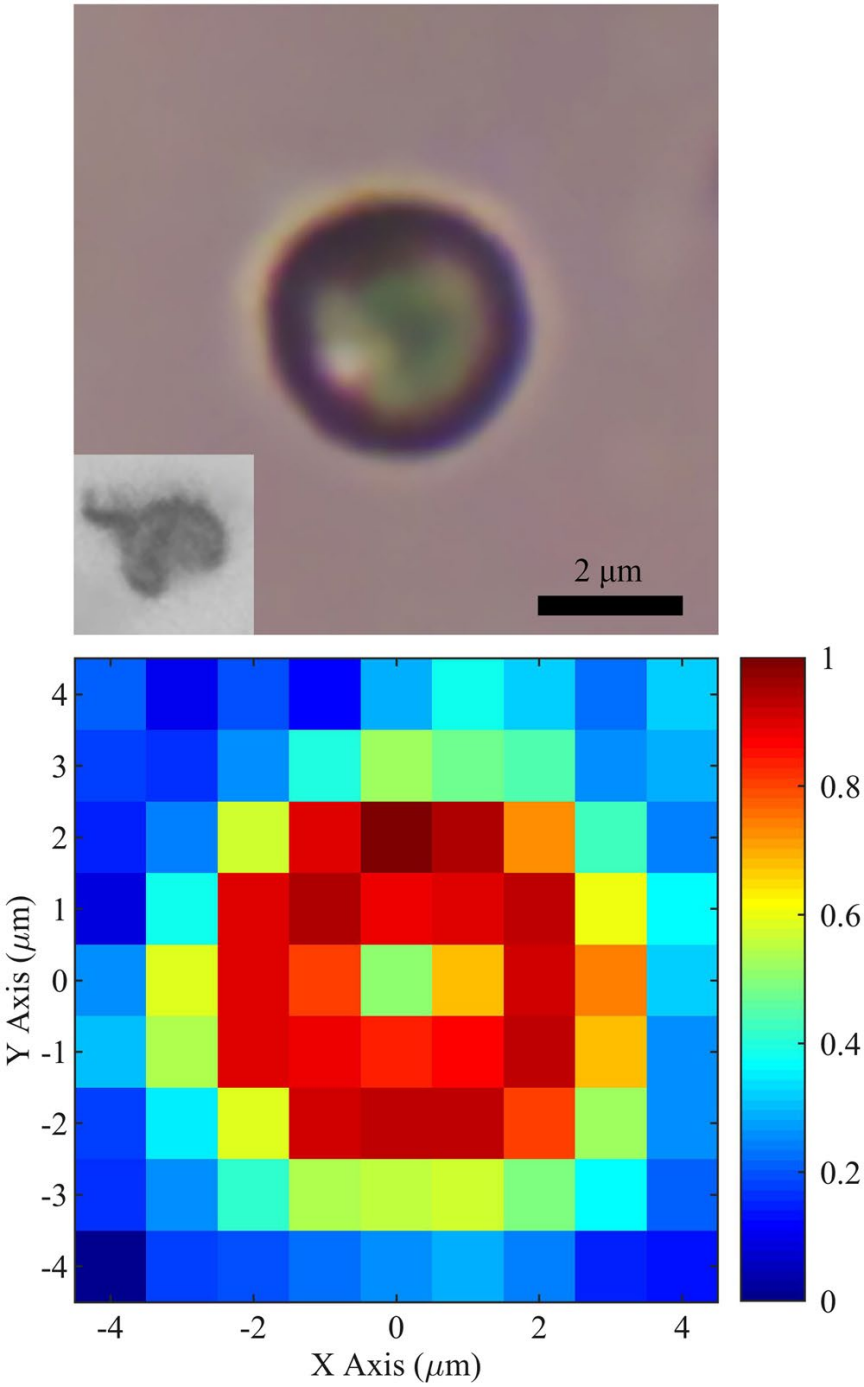
Top: optical image of an intact spore (conidium) of the fungus *A*. *nidulans*. The inset shows a lysed spore. Bottom: spectral similarity (calculated by the Pearson correlation coefficient) constructed from the average of 99 CARS maps of single, intact *A*. *nidulans* spores. The reference spectrum has coordinates (0,2) and is graphed in blue in Fig. 3. The spectrum of the middle of the spore with coordinates (0,0) is graphed in red in Fig. 3. Pixels surrounding the spore correspond to the glass substrate.

**Figure 3:**
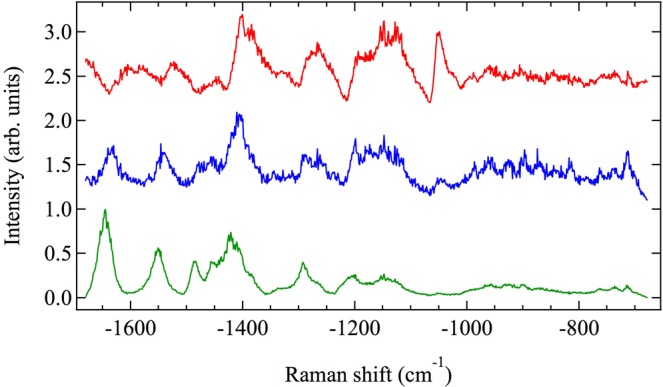
Corrected and averaged CARS spectra. Red and blue spectra belong to Fig. 2 (bottom) coordinates (0,0) and (0,2), respectively, and have been averaged over 99 intact spores. The green spectrum is the corrected and averaged CARS spectrum of 56 individual pixels from a total of 17 lysed spores and is the average CARS spectrum from the spore cell wall alone.

9 μm × 9 μm CARS maps were generated for each of 99 single, intact *A*. *nidulans* spores, each with a pixel resolution of 1 μm × 1 μm and an integration time of 1 s per CARS spectrum per pixel. The corrected spectra of each pixel were then averaged over the 99 spores in order to construct a single CARS map of an “average spore.” The result of this procedure is graphed in Fig. 2 (bottom), which shows the spectral similarity (calculated by the Pearson correlation coefficient) of each pixel’s averaged spectrum as referenced to the averaged spectrum of a point on the edge of the spore with coordinates (0,2). The reference spectrum on the edge of the spore is graphed in blue in Fig. 3, while the spectrum of the conspicuous pixel with coordinates (0,0) in the middle of the spore is graphed in red in Fig. 3. Both the red and the blue spectra in Fig. 3 are used as reference spectra in subsequent calculations.

**Figure 4:**
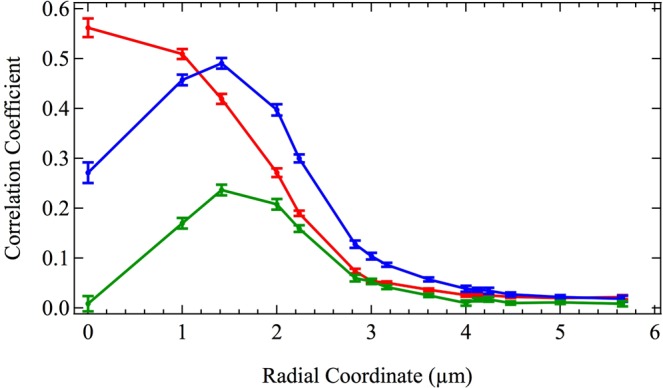
Spectral similarity of CARS spectra (calculated by the Pearson correlation coefficient) as a function of radius averaged over 99 intact spores. Red, blue, and green curves correspond to the reference CARS spectra shown in red, blue, and green in Fig. 3, respectively, which are the middle, edge, and cell wall of *A*. *nidulans* spores. Error bars show the standard deviation of the mean.

The relationship between the CARS spectra in the middle and edge of the spore becomes more apparent if the azimuthal symmetry of the spores is used to average the data in the radial direction, as well. The result of averaging in the radial direction is shown in Fig. 4, where the (0,0) coordinate in Fig. 2 (bottom) is taken as the zero radius. The red curve shows the average similarity of the CARS spectra measured at a given radius (for all 99 sampled spores) referenced to the red CARS spectrum in Fig. 3, which corresponds to the middle of the spore. The blue curve in Fig. 4 shows the average similarity at a given radius (again, for all 99 sampled spores) referenced to the blue spectrum in Fig. 3, which corresponds to the edge of the spore. The error bars show the standard deviation of the mean.

The similarity curves in Fig. 4, along with previously published transmission electron micrographs of *A*. *nidulans* spores^23^, suggest that CARS measurements taken in the middle of the spore correspond primarily to the cytoplasm, while CARS measurements taken at the edge of the spore correspond to the cell wall. In order to test this hypothesis, a sample of the spores was lysed with glass beads (see Materials and Methods, above) in order to fracture and separate the cell wall from the cytoplasm. The CARS spectra of the cell walls of 17 lysed spores were then measured, for a total of 56 CARS spectra. The average CARS spectrum of these 56 measurements of the cell wall of lysed spores is shown in green in Fig. 3. It is similar in shape to the spontaneous Raman spectrum of *A*. *nidulans* spores published by Farazkhorasani^9^, although there are minor differences because of the expected contribution from the non-resonant background of the CARS process^20–22^. This average CARS spectrum of the cell wall of the spores is used as a reference spectrum for the green similarity curve in Fig. 4, which shows the average similarity of the measured CARS spectra of the 99 intact spores as a function of radius. It is important to note that the similarity of the 99 spores calculated in reference to a CARS spectrum consisting only of the cell wall peaks at the edge of the spore and is zero at zero radius, indicating that CARS measurements taken in the middle of intact spores correspond primarily to the cytoplasm, while those taken at the edge of the spore correspond primarily to the cell wall.

The differences in the CARS spectra for the edge and center of the intact spores arise from the differing geometry and chemical compositions of these structures. When dry and dormant, the cell wall has three layers. The outermost layer is approximately 7–14 nm thick and is composed of hydrophobin protein rodlet structures^24,25^. The inner two layers are 126–252 nm and 140–224 nm thick, respectively, and are composed primarily of glucan and chitin^24,26,27^. Separating the layered cell wall from the inner cytoplasm is the cell membrane composed of a bilayer of lipids^28^. The cytoplasm contains the organelles of the cell as well as deposits of energy-rich molecules. Molecules found in the cytoplasm include nucleotides, proteins, polysaccharides, and lipids, among many others^24,29^.

Since the cell wall has a finite thickness ranging from 273–490 nm and surrounds the entirety of the cytoplasm, CARS spectra corresponding to the edge of the spore contain Raman contributions primarily from the cell wall. CARS spectra corresponding to the middle of the spore contain Raman contributions primarily from the cytoplasm, as mentioned above and shown in Fig. 4. The different ensembles of molecules contained in these two sampling regions of the spore result in the collection of Raman peaks shown in Fig. 3. The spectra are indicative of the total contribution from each of the many molecules contained in each sampling region. While the spectra therefore contain information about the compositions of the cell wall and the cytoplasm, respectively, in this context they function primarily as spectral fingerprints.

**Figure 5:**
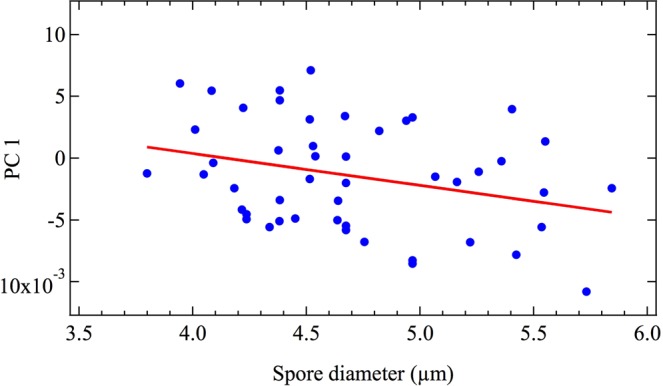
The primary PC scores of the average spectra of the edge of single spores of *A*. *nidulans* versus their minimum diameter. Data points are graphed in blue, while a linear fit is shown in red.

In order to investigate the ability of CARS spectroscopy to characterize spore heterogeneity, PCA was used to quantify the variation in the corrected CARS spectra of a subset (*n* = 48) of the above *A*. *nidulans* intact spores. Spores included in the subset had minimum diameters that were able to be measured using image analysis software (see Materials and Methods). The first PC score of the spectrum of the edge of each spore is graphed against its minimum diameter in Fig. 5. There is a weak but measurable CARS spectral variation with spore diameter, corresponding to a Pearson correlation coefficient of *ρ* = −0.302 with *R*^2^ = 0.091. In contrast, the calculated spectral variation of the center of each spore versus its diameter, which is not graphed, has a Pearson correlation coefficient of *ρ* = 0.044 with *R*^*2*^ = 0.002, which is about an order of magnitude less. From these calculations it is evident that there is a correlation between the spectrum of the cell wall and spore diameter, but not between the spectrum of the cytoplasm and the spore diameter. It can be concluded, then, that CARS spectroscopy can indeed provide a measure of spore heterogeneity, even when the spores under analysis are genetic clones of each other and are grown under the exact same conditions, as is the case here. It is to be expected then, that CARS may quantify even greater degrees of heterogeneity, such as those arising from disparate growth media and environments (see^30^, for example).

**Figure 6:**
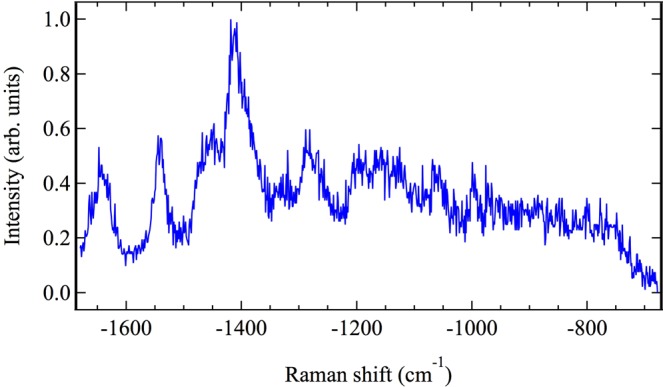
CARS spectrum of the cell wall of a single *Aspergillus nidulans* spore, taken on a sapphire substrate with an integration time of 0.1 s.

We have demonstrated that a transmissive CARS setup can rapidly measure and map fungal spores. While each measured CARS spectrum used in the above study was integrated for 1 s, we found that an integration time of 0.1 s was sufficient to obtain a recognizable *A*. *nidulans* CARS spectrum, as shown in Fig. 6. Here, the measured raw spectrum was not processed in any way, so that the background non-resonant signal remains. Nevertheless, the characteristic peaks that identify the spectrum as belonging to the cell wall are clearly evident. In comparison, additional attempts to measure the spontaneous Raman spectrum using a continuous wave laser with 1064 nm wavelength necessitated an integration time of at least 100 s or more to obtain a recognizable spectral shape. The advantage of CARS over spontaneous Raman for the development of a rapid spore identification technique is therefore obvious. Moreover, we have conclusively shown that the transmissive CARS scheme detailed here differentiates even between the cytoplasm and the cell wall of the spore itself. Not only does a CARS system have dramatic potential as a valuable tool for real-time identification of mold species, but it may also prove to be a powerful tool for mycologists to investigate biological processes which manifest differently within the cytoplasm and the cell wall of a fungal spore. We therefore predict that coherent Raman processes will find increased usage in the identification and study of fungi.

## Supplementary information

Supplementary Information

## Data Availability

The datasets generated during and/or analyzed during the current study are available from the corresponding author on reasonable request.
