# Supplementary material for: CARS spectroscopy of Aspergillus nidulans spores
Source: Sci Rep. 2019 Feb 11;9:1789. doi: 10.1038/s41598-018-37978-6 (PMC6370834; doi:10.1038/s41598-018-37978-6)
Supplement: Supplementary file 1 — Supplementary Information [file 41598_2018_37978_MOESM1_ESM.docx]

CARS spectroscopy of *Aspergillus nidulans* spores

Benjamin Strycker,^1,2^ Zehua Han,^1^ Blake Commer,^3^ Brian Shaw,^3^ Alexei Sokolov,^1,2^ Marlan Scully^1,2^

^1^Institute for Quantum Science and Engineering, Texas A&M University,

College Station, Texas, USA

^2^Baylor University, Waco, Texas, USA

^3^Department of Plant Pathology and Microbiology, Texas A&M University, College Station, Texas, USA

**APPENDIX 1: Beam Parameters**

The beam waists of the pump/probe and Stokes pulses of the CARS setup were measured with the knife-edge technique in the both the x- and y-directions. The edges used for measurement belonged to the square of the 2^nd^ element of the 6^th^ group of the USAF 1951 Resolution Test Target. Beam waists were measured as a function of the depth coordinate z, so as to measure the Rayleigh length of each beam, as well. The results are shown in Figure S1, with the pump/probe beam in blue and the Stokes beam in red. Solid and empty data markers correspond to measurements in the x- and y-directions, respectively. Also shown are fits for a Gaussian beam waist of the form $w\left( z \right)=w_{0}\sqrt{1+{(z/z_{R})}^{2}}$ in solid and dotted lines, which correspond to the x- and y-directions, respectively. Vertical lines show the measured Rayleigh lengths of each beam in the x- and y-directions. The pump/probe and Stokes beams fit well to Gaussian parameters. When spatially overlapped, the approximate beam waist is 2 μm, which, according to the Sparrow criterion for Gaussian beams, corresponds to a spatial resolution of 1 μm.


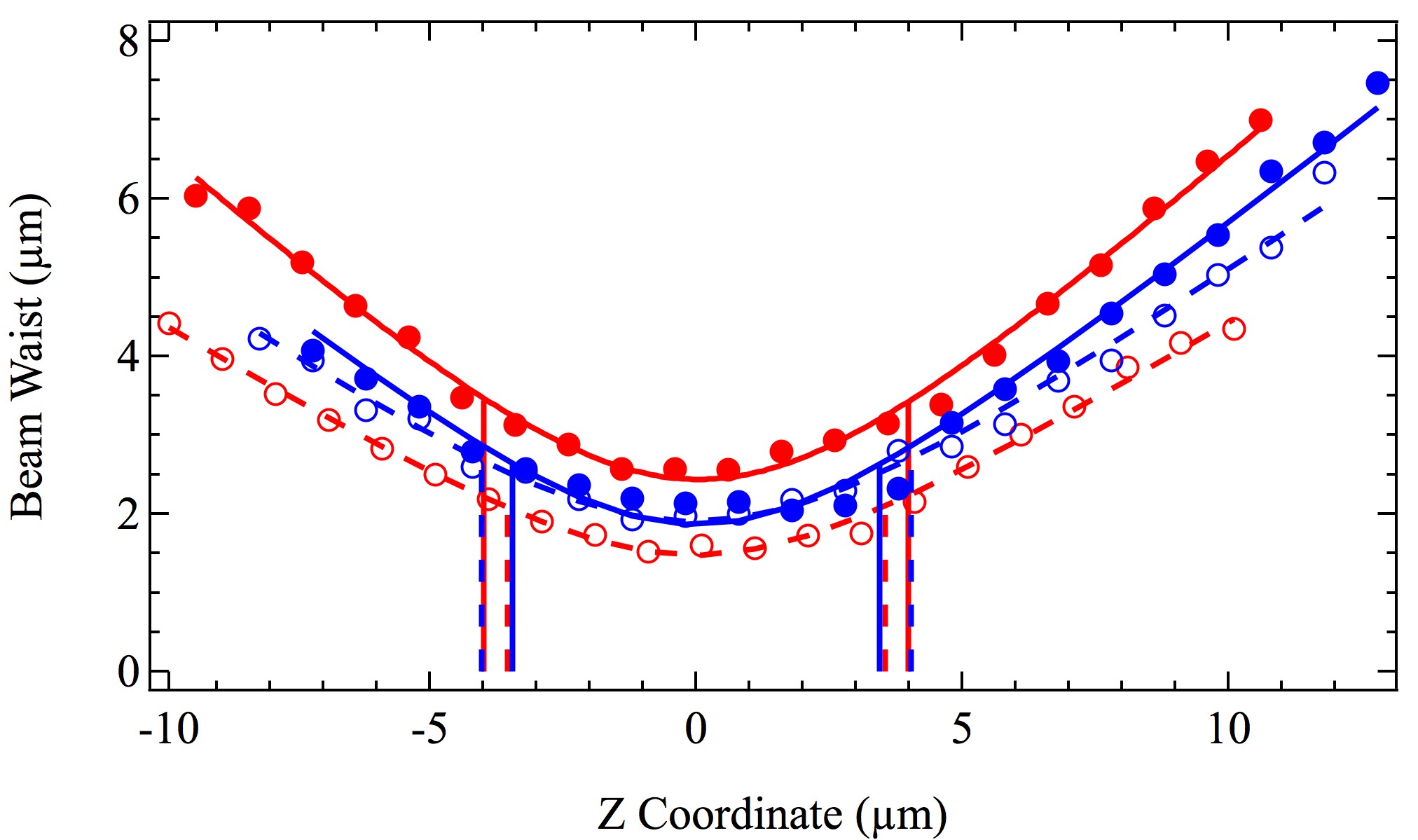


Figure S1. Beam waist of the pump/probe (blue) and Stokes (red) beams used in the experiment. Solid and empty markers correspond to measurements in the x- and y-directions, respectively, as do solid and dotted theoretical Gaussian waist fit lines. The vertical lines show the measured Rayleigh length of each beam.

**APPENDIX 2: Signal strength as a function of time**

In order to find a suitable beam power at which to take CARS measurements of *A. nidulans* spores, the integrated CARS spectrum (as calculated using the scheme in Materials and Methods above) was measured over a period of approximately 300 seconds for three different beam powers. The results are shown in Figure S2. The top, middle, and bottom curves correspond to beam powers of: 2.25 mW for the pump/probe and 1.2 mW for the Stokes; 1.65 mW for the pump/probe and 0.95 mW for the Stokes; and 1.07 mW for the pump/probe and 0.70 mW for the Stokes, respectively. Each curve is the averaged response of five individual spores. For each beam power level, the signal power remains relatively constant for about 10 s, after which the sample begins to experience thermal degradation, and the signal decreases. All of the CARS measurements used in this study were integrated for 1 s using a beam power of 1.65 mW for the pump/probe and 0.95 mW for the Stokes beam (corresponding to the middle curve). Consequently, our measurements do not suffer from thermal degradation of the sample. We note that the CARS signal strengths at t = 0 for each beam power level are consistent with a saturation model in the presence of strong absorption in the sample [31].


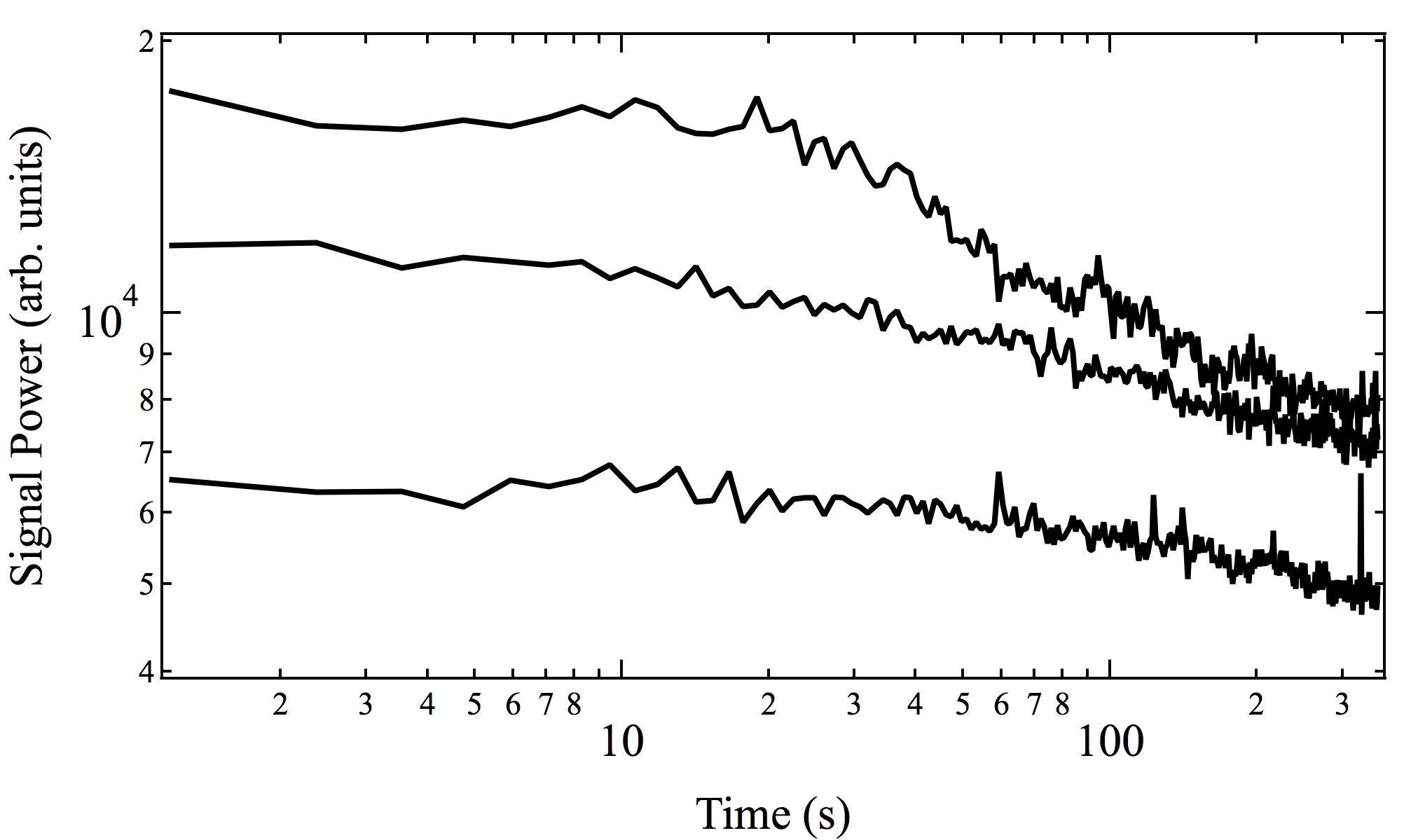


Figure S2. Integrated CARS signal power as a function of time. The top, middle, and bottom curves correspond to beam powers of: 2.25 mW for the pump/probe and 1.2 mW for the Stokes; 1.65 mW for the pump/probe and 0.95 mW for the Stokes; and 1.07 mW for the pump/probe and 0.70 mW for the Stokes, respectively.

**REFERENCES**

# [31] Zhi, M., *et al.* Concentration dependence of femtosecond coherent anti-Stokes Raman scattering in the presence of strong absorption. *J. Opt. Am. B* 24(5), 1181-1186 (2007).
